# Evaluation of the Direct and Indirect Regulation Pathways of Glutathione Target to the Hepatotoxicity of Microcystin-LR

**DOI:** 10.1155/2018/5672637

**Published:** 2018-06-27

**Authors:** Wan-song Zong, Shu-han Zhang, Qian Wang, Yue Teng, Yu-zhen Liu, Yong-gang Du

**Affiliations:** ^1^College of Geography and Environment, Shandong Normal University, Jinan 250014, China; ^2^School of Environmental and Civil Engineering, Jiangnan University, Wuxi 214122, China

## Abstract

Glutathione (GSH) plays crucial roles in regulating the hepatotoxicity of Microcystin-LR (MCLR) by inhibiting oxidative stress or by toxin conjugation. Based on MCLR conjugation product preparation and purification, the direct and indirect regulation pathways for GSH were fully evaluated. Protein phosphatase inhibition analysis verified that GSH conjugation was an effective pathway to regulate the inhibition effect of MCLR, while GSH had slight influence on the toxicity of MCLR. Research on oxidative stress showed that both regulation pathways could reduce the formation of reactive oxygen species (stimulated by MCLR and regulated by NADH oxidase) and regulate the adverse effects on antioxidant enzymes. By evaluating the contributions for both pathways, it could be found that the indirect pathway had significant contribution to eliminating cellular reactive oxygen species and regulating protein phosphatases inhibition, while the direct regulation pathway had moderate influence. As glutathione transferases facilitated the transformation of MCLR, the hepatotoxicity of MCLR could be effectively regulated by GSH conjugation pathway, especially with abundant exogenous GSH.

## 1. Introduction

Microcystins (MCs) are naturally occurring toxic metabolites produced by bloom-forming genera of cyanobacteria and are released to the water phase at cell lysis [1, 2]. MCs present health risks worldwide for humans and wildlife that drink or use contaminated water. Orally ingested, MCs are transported into the bloodstream via the ileum and tend to be absorbed by hepatocytes, causing disintegration of hepatocyte structure, apoptosis, liver necrosis, and hemorrhagic shock [3]. Furthermore, MCs are suspected of promoting primary liver cancer with long-term exposure to sublethal levels [4].

Within the hepatocytes, the main mechanism of MCs toxicity is associated with the specific inhibition of protein phosphatase 1 and 2A (PP1 and PP2A), leading to increased phosphorylation of key proteins involved in signal transduction. Defective protein phosphorylation/dephosphorylation induced cytoskeleton disorganization and cell integrity disruption [5]. There is also substantial evidence that MC-dependent toxicity is accompanied by oxidative stress in hepatocytes [6]. The production of reactive oxygen species (ROS) results in an increase in lipid peroxidation, the alteration of antioxidant defense system, and DNA damage. There are two possible explanations for the source of ROS with MCs pollution: ROS can be generated by stimulating NADH oxidase or by inhibiting PP1/PP2A activity [4].

Accordingly, regulations on the biological toxicity of MCs mainly included oxidative stress inhibition and detoxification metabolism. As the typical feature for oxidative stress is generated ROS surpassing the removal ability of antioxidant system, the hepatotoxicity of MCs could be regulated by enhancing the antioxidation capacity of hepatocytes. Researchers have evaluated the inhibition effect of antioxidants vitamin, GSH, and sulforaphane on MCs induced oxidative stress [7, 8]. Related results verified that the oxidative stress levels of antioxidants intervention groups had certain degrees of decline compared with control groups. MCs detoxification metabolism mainly involves glutathione transferases (GSTs), which are in the first line of molecular defenses against environmental stressors [9]. With the catalysis of GSTs, GSH participates in the metabolic process of MCs and the formation of MC-GSHs appears to be the key for MCs detoxication.

In view of the critical detoxification effect of GSH, the hepatotoxicity of MCs could be regulated by a direct pathway (direct inhibition of oxidative stress) or by an indirect pathway (forming MC-GSH). However, there are several problems to be solved for further clarifying the detoxification mechanism of GSH: (1) in hepatocytes, the newly formed MC-GSHs usually coexist with original toxins and the independent regulation effect for GSH conjugation pathway is difficult to determine; (2) although oxidative stress could be regulated by GSH in direct and indirect pathways, the specific contribution for each pathway is unknown; (3) there is no commercial standard for MC-GSHs, which also restricts above studies toward elucidating the regulation mechanism of GSH.

To better understand the direct and indirect regulation strategies of GSH target to MCs, MCLR-GSH originating from MCLR (a typical MC) were synthesized through electrophilic addition reaction and purified according to the traditional method for MCLR [10]. Then, the biological toxicity of MCLR and MCLR-GSH was evaluated to explore the regulation effect of GSH transformation for PP1/PP2A and cellular PPs activity. Subsequently, oxidative stress level in hepatocytes (treated with MCLR, MC-GSH, and MCLR+GSH) was investigated to verify the mechanism and effectiveness for the two regulation pathways. By investigating the activity of GSTs, the influence of MC-GSH transform on cellular PPs activity and oxidative stress level was discussed to quantify the contribution for both pathways.

## 2. Materials and Methods

### 2.1. Materials

MCLR standard was purchased from Sigma (Saint-Quentin-Fallavier, France). HPLC acetonitrile, methanol, and trifluoroacetic acid were obtained from Merck (Darmstadt, Germany). PP1 (1500 U/mL) from rabbit skeletal muscle and PP2A (catalytic subunit, 100 U/mL) from human red blood cells were obtained from EMD Millipore (Darmstadt, Germany). Reactive oxygen species (ROS), NADH oxidase (NOX), superoxide dismutase (SOD), catalase (CAT), and glutathione S-transferase (GST) assay kits were purchased from Nanjing Jiancheng Bioengineering, Inc. (China). Other regents were purchased from Sinopharm (Shanghai, China) and were of analytical or higher grades.

### 2.2. MCLR-GSH Identification and Preparation

#### 2.2.1. Addition Reaction for MCLR-GSH

In order to prepare MCLR-GSH, 2 *μ*M MCLR and 500 *μ*M GSH were mixed in 5% K_2_CO_3_ and incubated for 2 h at room temperature [11]. The reaction mixture was neutralized with 0.2 M HCl and applied to conditioned Cleanert C_18_ SPE cartridges (500 mg, Bonna-Agela) that were rinsed with 10 mL methanol and 15 mL water. The impurities were eluted with 10 mL 10% methanol and MCLR and MCLR-GSH were eluted with 10 mL 80% methanol. The eluted samples were evaporated to dryness in N_2_ flow and resuspended in 1 mL acetonitrile. The prepared samples were stored in -20°C before analysis.

#### 2.2.2. MS and MS/MS Analysis of MCLR and MCLR-GSH

The crude extract for MCLR-GSH was analyzed by the maXis UHR-TOF mass spectrometer (Bruker Daltonics). Samples were mixed with isometric acetonitrile (containing 0.1% trifluoroacetic acid) and injected into MS spectrometer at 5 *μ*L/min. MS parameters were set as follows: positive ion mode, electrospray source voltage 4.0 kV, cone voltage 0.4 kV, desolvation gas N_2_ 0.6 bar, dry gas N_2_ 4.2 L/min, dry gas heater 180°C, and scan range 400-1500 [12]. Data acquisition was controlled with the Compass software and MCLR/MCLR-GSH could be detected according to their m/z signals. MCLR-GSH was further identified by comparing its specific secondary ions with that of MCLR. MS/MS parameters were set as that of MS analysis except that N_2_ collision gas was used and collision energies were adjusted at 40 eV.

#### 2.2.3. MCLR-GSH Preparation

Resuspended samples containing MCLR and MCLR-GSH were further separated using a Great Eur-Asia C_18_ column (9.4 × 250 mm, 5 *μ*m, 120 Å) on an Alliance 2695 HPLC system prior to MS analysis. The injection volume was 100 *μ*L and the mobile phase was a gradient elution of water (mobile phase A) and acetonitrile (mobile phase B), both containing 0.1% formic acid. The gradient elution was programmed as follows: 0-5 min, 20%B; 35-40 min, 80%B; and 40.1-45 min, 20%B (35°C, 2 mL/min) [13, 14]. MS parameters were set as Section 4.2.2. Purified MCLR-GSH was manually collected according to its specific retention time, evaporated to dryness with N_2_, and dissolved in 200 *μ*L methanol. Finally, MS analysis of isolated MCLR-GSH was performed to evaluate its concentration and purity with MCLR standard as reference.

### 2.3. Protein Phosphatase Inhibition Assay

The inhibition of toxins on PP1 and PP2A was evaluated by a colorimetric protein phosphatase inhibition assay [12]. PP1 and PP2A were diluted to 5 U/mL with freshly prepared buffers. The assay was conducted by addition of 10 *μ*l PP1/PP2A to 100 *μ*l test samples in a 96-well polystyrene microplate. With gentle shaking, the microplates were incubated at 37°C for 10 min and p-nitrophenyl disodium orthophosphate was added. After 1 h, the absorbances (p-nitrophenol production) of incubated samples were measured in a Thermo/max microplate reader. The inhibition of test samples on PPs could be expressed as follows:

(1) $$\begin{array}{c} I_{PPs}=\frac{A_{control}-A_{sample}}{A_{control}}\times 100\% \end{array}$$

where A_control_ and A_sample_ were the absorbance of reference sample (without PPs) and test sample at 405 nm, respectively.

### 2.4. Cellular Toxicity Assay

#### 2.4.1. Cell Culture and Exposure

HepG2 (human hepatocellular liver carcinoma cell line) was obtained from Cell Bank of Chinese Academic of Science and grown in DMEM medium supplemented with 10% (v/v) fetal bovine serum, 100 unit/mL penicillin, and 100 *μ*g/mL streptomycin at 37°C and 5% CO_2_. Whenever cells reached about 90% confluence, they were detached, reconstituted with medium, and split into the required potions for the next seeding stage. Then HepG2 cells were treated with MCLR (with or without GSH) and MCLR-GSH for 6-48 h.

#### 2.4.2. Biochemical Analysis

The contents of cellular ROS and the activities of PPs, SOD, CAT, NOX, and GST were assayed by the kits purchased from Nanjing Jiancheng Bioengineering, Inc. (China). All the procedures were followed according to the manufacturer's instructions. In brief, ROS was evaluated by 2,7-dichlorofluorescein diacetate assay. PPs activity was measured using the method described by Li et al. [15]. SOD activity was measured using the method described by Giannopotitis and Ries [16]. CAT activity assay was performed following the method of Cakmak and Marschner [17]. NOX activity was evaluated by 2,6-dichloroindophenol assay. NOX catalyzed the oxidation of nicotinamide adenine dinucleotide (NADH) and formed NAD^+^. When 2,6-dichloroindophenol (in the blue colour) was introduced, the oxidation of NADH correlated to the reduction of 2,6-dichloroindophenol (forming colorless product). By measuring the characteristic absorbance for 2,6-dichloroindophenol at 600 nm, the enzyme activity for NOX could be calculated out. GST activity was detected by evaluating the conjugation of GSH with the standard model substrate 1-chloro-2,4-dinitrobenzene [18]. Cellular protein contents were assayed according to the method described by Bradford, using bovine serum albumin as the standard [19].

#### 2.4.3. Data Analysis

Each assay was carried out in triplicate to obtain means and standard deviations.

## 3. Results and Discussion

### 3.1. MCLR-GSH Identification and Preparation

**Figure 1:**
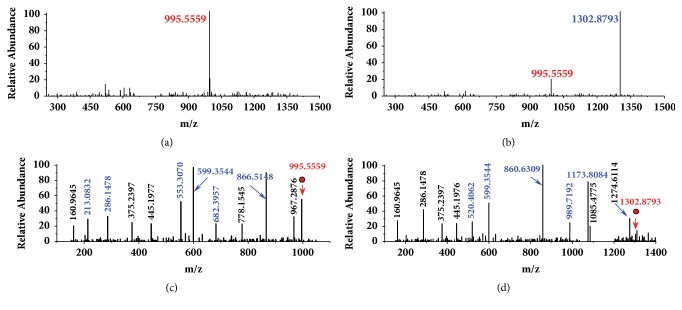
MS analysis for MCLR and its electrophilic addition product MCLR-GSH. Conditions: MS spectra for MCLR (a) and its electrophilic addition sample (b); MS/MS spectra for MCLR (c) and MCLR-GSH (d).

Conjugated with GSH, MCs transform into specific MC-GSHs with different molecular weight (+307.32348 Da) that could be probe by mass spectrograph. For MCLR with a molecular weight of 994.5482 Da (Figure 1(a)), its primary MS signal was detected at m/z 995.5559, corresponding to the single-proton product. For GSH conjugation sample, MCLR still exist in mass spectrum but had lower intensity than the newly formed ion with MS signal at m/z 1302.8793 (Figure 1(b)). Undoubtedly, the signal should be attributed to MCLR-GSH, the addition product of GSH to MCLR. The generative mechanism for MCLR-GSH was confirmed by comparing its secondary structures with MCLR [11]. MS/MS analysis for MCLR showed that typical CID fragments were detected at m/z 213.0832, 286.1478, 553.3070, 682.3957, and 866.5148 (Figure 1(c)), corresponding to the secondary structures of [Glu-Mdha+H]^+^, [MeAsp-Arg+H]^+^, [Mdha-Ala-Leu-MeAsp-Arg+H]^+^, [Arg-Adda-Glu-Mdha+H]^+^, and [Mdha-Ala-Leu-MeAsp-Arg-Adda+H]^+^/[Arg-Adda-Glu-Mdha-Ala-Leu+H]^+^ [13]. Based on the same strategy, the CID fragments for MCLR-GSH were also obtained (Figure 1(d)). MCLR-GSH had partial identical fragment ions as that of MCLR (e.g., 160.9645 and 286.1478). It also had several newly formed CID fragments at m/z 520.4062, 860.6309, 989.7196, and 1173.8084, corresponding to [Glu-Mdha+H]^+^+307.3240, [Mdha-Ala-Leu-MeAsp-Arg+H]^+^+307.3239, [Arg-Adda-Glu-Mdha+H]^+^+307.3239, and [Mdha-Ala-Leu-MeAsp-Arg-Adda+H]^+^/[Arg-Adda-Glu-Mdha-Ala-Leu+H]^+^+307.3236. It was not difficult to find that the mass change (307.3238 ± 0.0002) was related to Mdha^7^ residue in MCLR. In accordance with literature, GSH was added to the C=C bond of Mdha^7^ residual [11].

### 3.2. Influence of MCLR, MCLR-GSH, and GSH on PP1, PP2A, and Cellular PPs Activity

To evaluate and compare the potential toxicity of MCLR-GSH with MCLR, MCLR-GSH was purified with SPE and preparative chromatography techniques. The preparation and purification information was listed in Table 1. As MCLR-GSH had higher concentrations and purity, the prepared sample could be used to evaluate its toxicity at molecular and cellular levels.

**Figure 2:**
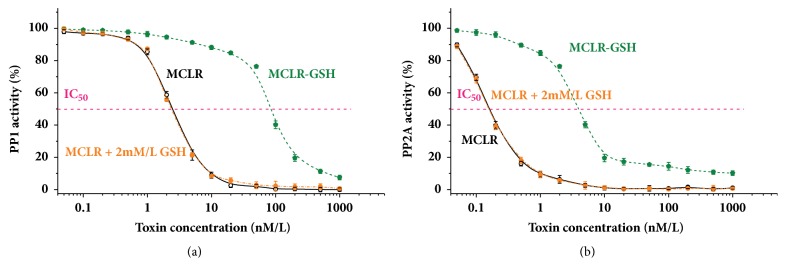
Inhibition curves for MCLR, MCLR+2 mM/L GSH, and MCLR-GSH on PP1 (a) and PP2A (b).

The inhibition effects of MCLR and MCLR-GSH on PP1/PP2A were compared to clarify the regulation effect of GSH conjugation at molecular level. According to PPs inhibition assay, the inhibition curves for MCLR (with or without GSH) and MCLR-GSH were plotted and specific IC_50_ values were calculated out. Compared with the native toxin, MCLR-GSH had much lower inhibiting effect on PP1 (IC_50_ 2.31 *μ*g/L versus 84.42 *μ*g/L), indicating that GSH conjugation was an effective pathway to control the toxicity of MCLR target to PP1 (Figure 2(a)). To better understand the regulation effect of GSH conjugation, the activity of PP1 exposed to MCLR and physiological level GSH (2 mM/L) was also obtained (IC_50_ = 2.23 *μ*g/L). However, experiment data showed that the addition of GSH had slight impact on the inhibition effect of MCLR target to PP1. GSH conjugation was also an effective pathway to control the inhibition effect of MCLR target to PP2A (Figure 2(b)). IC_50_ value for MCLR and MCLR-GSH was 0.16 and 4.10 *μ*g/L, respectively. When 2 mM/L GSH was added to the test samples, the inhibition effect of MCLR target to PP2A also showed unobvious difference (IC_50_ = 0.15 *μ*g/L).

**Figure 3:**
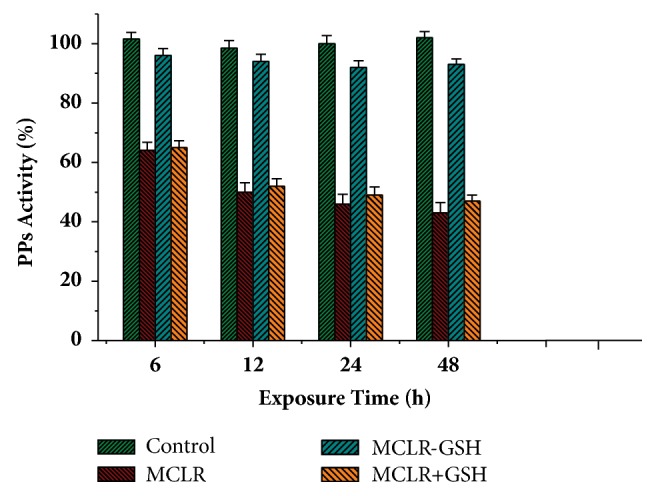
Effect of MCLR, MCLR+GSH, and MCLR-GSH on the activity of intracellular PPs. Conditions: MCLR 20 nM/L, MCLR-GSH 20 nM/L, and GSH 2 mM/L. PPs activity for control sample at 0 h was set as 100%.

Based on molecular toxicity test for the activity of PP1/PP2A, the Influence of MCLR and MCLR-GSH on cellular PPs activity was further evaluated (Figure 3). For control samples, the activity for cellular PPs was slightly changed with the increase of cultivation time. Treated with 20 nM/L MCLR (a typical toxin concentration for cyanobacterial blooms), the activity for cellular PPs was significantly inhibited. Compared with MCLR, MCLR-GSH had much weaker inhibition effect on cellular PPs: with the extension of exposure time, MCLR-GSH gradually showed certain inhibition effect (the activity for PPs merely decreased by 7% after 48 h). Undoubtedly, GSH conjugation was an effective pathway to regulate the hepatotoxicity of MCLR. However, the residual toxicity for transformed MCLR-GSH was also worth of consideration. In addition to the activities declining slightly slowly, the change trend for cellular PPs activity was consistent with the action of MCLR. The diversity for cellular PPs activity should be attributed to the conjugation of GSH with partial MCLR. To conclude, the transformation of MCLR to MCLR-GSH had significant influence on PPs activity and was an effect pathway to regulate the toxicity of MCLR.

### 3.3. Influence of MCLR, MCLR-GSH, and GSH on Cellular Oxidative Stress in Hepatocytes

**Figure 4:**
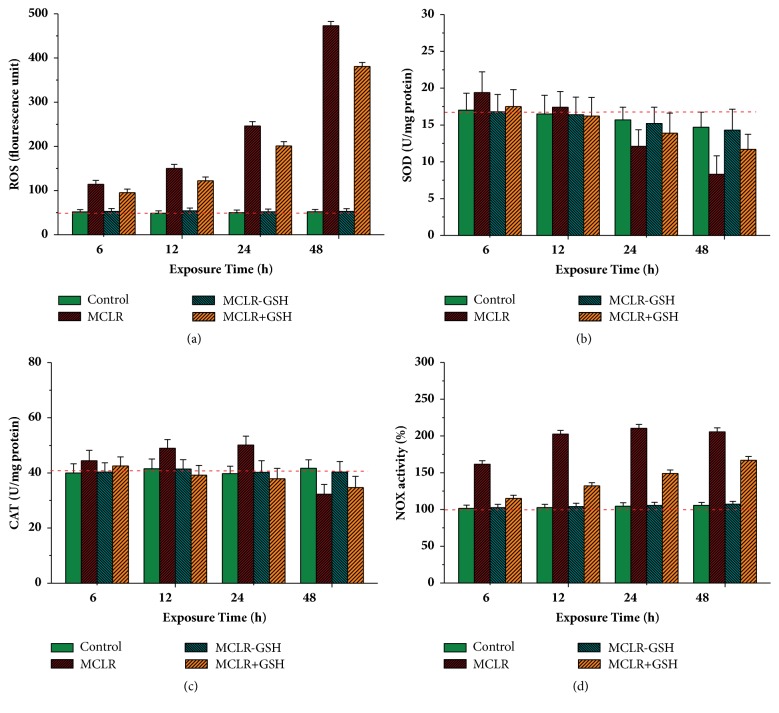
Influence of MCLR, MCLR+GSH, and MCLR-GSH on cellular ROS content (a) and catalase (b), superoxide dismutase (c), and NOX (d) activities in HepG2 cells. Conditions: MCLR 20 nM/L, MCLR-GSH 20 nM/L, and GSH 2 mM/L; the dash lines correspond to the original data for control samples at 0 h; original NOX activity for control sample was set as 100%.

Cellular oxidative stress induced by MCs was usually accompanied by raised ROS and the changed activities of antioxidant enzymes SOD, CAT, and so forth [3, 6]. For MCs toxicity regulation, above indexes could be used to evaluate the direct and indirect regulation effects of GSH. To evaluate the influence of toxins on oxidative stress level, cellular ROS in HepG2 cells was firstly measured (Figure 4(a)). For control samples, the content of ROS was slightly affected by incubated time. Treated with MCLR, the content of cellular ROS increased by a large margin, indicating that MCLR had significant stimulation on the Redox equilibrium of HepG2 cells. Compared with the original toxin, MCLR-GSH had much weaker influence on cellular ROS level: there was no difference between samples with or without MCLR-GSH in statistics. When simultaneously treated with MCLR and GSH, the content of ROS increased to a great extent but was lower than samples singly incubated with MCLR. Accordingly, the direct regulation pathway had moderate influence on the level of cellular oxidative stress, while the indirect pathway had significant influence.

Based on ROS analysis, the influence of toxins on cellular antioxidant enzymes SOD and CAT was also investigated (Figures 4(b)-4(c)). For control samples, the activity for CAT was slightly changed, while the activity for SOD showed certain downward trend. Treated with MCLR, the activities for SOD and CAT both showed increased trends and then were changed into decreased trends. Besides, SOD also had apparent downward tendency compared to that of CAT. A possible explanation was that short time stimulation of MCLR was beneficial to enhancing cellular antioxidant enzyme activity to eliminate excess ROS. However, with ROS accumulated over extended incubation time, inhibition effects on these enzymes were gradually revealed. SOD had a peak of activity at about 6 h, while CAT had a peak of activity at about 24 h, meaning that SOD was much sensitive to the adverse effect of MCLR. For samples treated with MCLR-GSH, the change trends of cellular SOD and CAT activities were similar to those of control samples. However, transformation product had weaker influence on antioxidant system. When simultaneously treated with MCLR and GSH, though the enzyme activities for SOD and CAT showed increased trends before decreased trends, they both had peak activities at about 6 h. Compared with samples treated with MCLR, the decreased trends were much gentle. To conclude, reducing agent GSH and conjugation product MCLR-GSH could regulate the adverse effects of MCLR on antioxidant enzymes SOD and CAT.

As oxidative stress is directly related to NOX stimulation, clarifying the influence of toxins on NOX contributed to revealing the regulation mechanism for both pathways of GSH. For control samples, the enzyme activity for NOX showed certain upward trend with the extension of incubated time (Figure 4(d)). Treated with MCLR, though the activity for NOX evidently increased by about 60-110%, the increased tendency was still much lower than that of ROS (increased by about 121-800%, compared with Figure 4(a)). Comparatively, MCLR-GSH had similar increased trend to that of control samples, indicating that it had weaker influence on NOX. Treated with MCLR and GSH, the activity for NOX increased to a large extent but was lower than samples incubated with MCLR. As MCLR had significant stimulation to NOX and MCLR-GSH had weak influence, the decreased trend for NOX activity (for MCLR and GSH treated samples) should be attributed to the transformation of partial MCLR into MCLR-GSH. Beside the direct reduction action of GSH, the extra increment for cellular ROS should be attributed to the secondary effect of PPs inhibition.

### 3.4. Evaluation of the Contributions of the Direct and Indirect Regulation Pathways of GSH

Although research on PPs inhibition and oxidative stress showed that the direct and indirect regulation pathways of GSH both contributed to eliminating the adverse effects of MCLR, in most cases, the newly formed MC-GSHs coexist with original toxins and the contributions of the direct and indirect regulation pathways are difficult to determine. To evaluate the contributions of both regulation pathways of GSH, the change trends for cellular PPs activity and ROS content under different transformation degrees of MCLR were evaluated (the total concentration for MCLR and MCLR-GSH was set at 20 nM/L).

**Figure 5:**
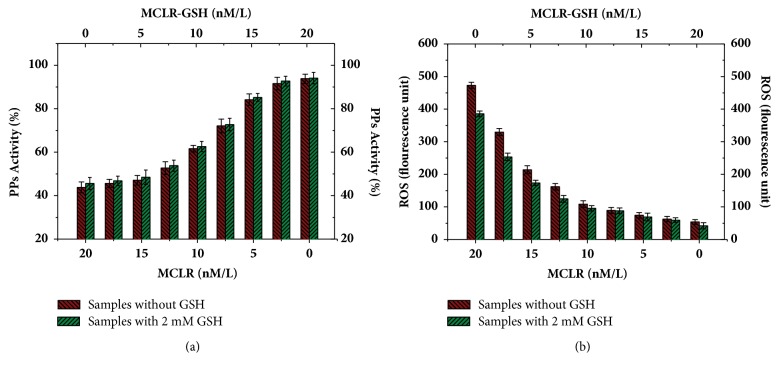
Cellular PPs activities (a) and ROS content (b) for HepG2 cells treated with different concentration of toxins. Conditions: PPs activity for control sample was set as 100%; incubated time was 48 h.

Figure 5(a) showed the contributions of the direct and indirect regulation pathways on cellular PPs activity. With the action of MCLR alone, cellular PPs activity was markedly inhibited and was about 43.8% as that of control sample. When MCLR was gradually changed into MCLR-GSH, cellular PPs activity showed evident increment trend. For the independent effect of MCLR-GSH, PPs activity increased to 93.6% after 48 h. Evidently, GSH conjugation was an effective pathway to regulate the cellular toxicity of MCLR. When physiological level GSH was introduced, the change trend for the activity of PPs was consistent with the combined action of MCLR and MCLR-GSH except that the activity was increased in certain degrees (0.3%-3.8%). Compared with the direct regulation pathway of GSH, GSH conjugation pathway had much significant contribution to reducing the inhibition effect of MCLR on PPs.

Figure 5(b) showed the contributions of the direct and indirect regulation pathways on cellular ROS content. For the independent effect of MCLR, the content of ROS increased largely (9.27 times of control sample). When MCLR was substituted by MCLR-GSH, the content of ROS decreased progressively. For the independent effect of MCLR-GSH, the content of ROS was slightly higher than that of control samples (1.04 times of control sample). Undoubtedly, GSH conjugation was also conducive to inhibiting oxidative stress. When simultaneously treated with GSH and MCLR/MCLR-GSH, the change trend for ROS content was also consistent with the combined action of MCLR/MCLR-GSH. But the content of ROS decreased by 2.3%-23.5% as that of samples treated without GSH. Compared with the direct regulation pathway of GSH, the indirect pathway also had much significant contribution to eliminating cellular ROS in HepG2 cells.

**Figure 6:**
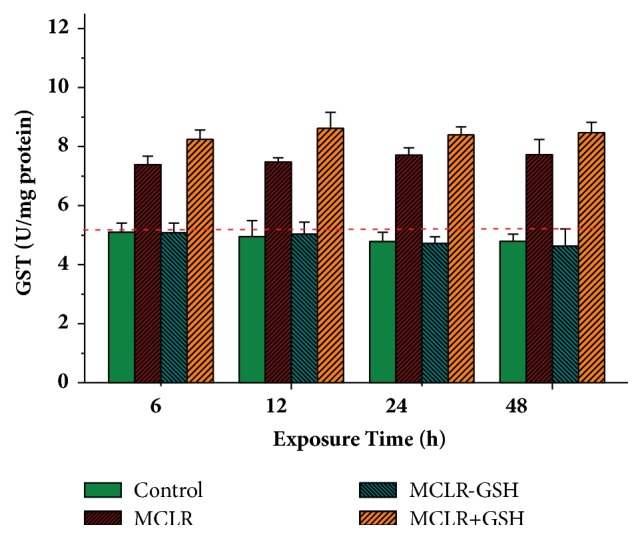
Influence of MCLR, MCLR+GSH, and MCLR-GSH on cellular GST activity in HepG2 cells. Conditions: MCLR 20 nM/L, MCLR-GSH 20 nM/L, and GSH 2 mM/L; the dash lines correspond to the original data for control samples at 0 h.

With the transformation of MCs to MC-GSHs involved in GST, the enzyme activity for GST was further used to evaluate the detoxification mechanism for indirect regulation pathway.  Figure 6 showed the influence of toxins on cellular GST activity. For control samples, GST activity showed certain downward trend. Treated with MCLR, the enzyme activity for GST showed evidently increased trend, indicating that the stimulation of MCLR was beneficial to enhancing the activity of GST to eliminate the toxin. For HepG2 cells treated with MCLR-GSH, the downward trend of cellular GST activity was similar to that of control samples. Undoubtedly, GSH conjugation product had no stimulation effect on GST. When simultaneously treated with MCLR and GSH, the enzyme activity for GST also showed increased trend. Due to the extra introduced GSH, enzyme activity for GST was slightly higher than that of single MCLR. Above results showed that GST facilitated the transformation of MCLR, especially with abundant exogenous GSH.

## 4. Conclusions

The hepatotoxicity of MCs could be regulated by directly inhibiting oxidative stress or by forming GSH conjugation products. Based on MCLR-GSH preparation and purification, the independent regulation effect for GSH conjugation pathway and the specific contribution for each pathway on oxidative stress were evaluated. By comparing the influence of MCLR-GSH and MCLR on PPs activity and cellular oxidative stress, it could be found that GSH conjugation pathway had significant influence on the hepatotoxicity of MCLR. When GSH was simultaneously introduced with MCLR, the PPs activity was slightly influenced and cellular oxidative stress was moderately influenced. Evaluation of the contributions of both regulation pathways indicated that the indirect pathway had much significant contribution for eliminating the adverse effect of MCLR. When MCLR was introduced, the activity of GST increased as well, indicating that GST might promote the transformation of MCLR by GSH conjugation pathway. This study offers a comprehensive cognition on MCs toxicity regulation and provides valid theoretical support to control their hepatotoxicity and environmental risk.

## Figures and Tables

**Table 1:** Preparation and purification information for MCLR-GSH.

Preparation product	Eluted time	Concentration	Total volume	Purity^b^
MCLR-GSH	12.52 ± 0.25 min	≈1200 *μ*mol/L^a^	5*∗*200 *μ*L	≈96.8%
MCLR standard	18.33 min	-* *-* *-	-* *-* *-	98.5%

^a^With 200 *μ*mol/L MCLR served as the inner standard for quantification and assumed MCLR and MCLR-GSH had approximate protonated efficiencies. ^b^Sample purity was directly calculated by MS signal intensity and defined as MCLR-GSH/(MCLR+MCLR-GSH) *∗* 100%.

## Data Availability

About the data availability statement, we have the following explanation. First of all, toxicity experiments were measured directly by us. The experimental procedure was described in the article. Then molecular simulation docking experiments were performed with Molecular Operating Environment software (MOE, version 16.09). The original model for MCLR-PP1 was obtained from Protein Data Bank (PDB code 1FJM, http://www.rcsb.org/pdb/home/home.do). Models for MCLR and PP1 were extracted based on the structure of MCLR-PP1. Models for MCLR transformation products were prepared based on the structure of MCLR. Before calculations, receptor PP1 was correct, protonated by adding hydrogen atoms, and small molecule ligands were minimized for energy optimization [19]. Then the interactions between toxins and PP1 were simulated and the experiment conditions were set as follows: Amber 10 EHT; Solvation R-Field; reaction temperature 25.0°C; pH 7.4; and salt 0.05 M. The key parameters such as the total energies, total combination areas, combination areas, hydrogen-bonds, and ionic bonds for the complexes and the main interaction sites were obtained for clarifying the detoxification mechanism of MCLR transformation pathway by MOE software. Software steps can be obtained from the authors. The correlation between toxin toxicity and hydrogen bonds and ion bonds for main interaction sites was evaluated by IBM SPSS statistics (version 19). Finally, Molecular Operating Environment software (MOE, version 16.09) was bought from a software company, so we are sorry that we cannot provide it to readers.
